# Pericardial effusion with a ruptured mycotic pseudoaneurysm secondary to *Salmonella* infection

**DOI:** 10.1590/0037-8682-0053-2022

**Published:** 2022-04-29

**Authors:** Lai Kuan Leong, Bui Khiong Chung, Chen Ting Tan

**Affiliations:** 1 Ministry of Health Malaysia, Miri General Hospital, Department of Medicine, Miri, Sarawak, Malaysia.; 2 Ministry of Health Malaysia, Sarawak General Hospital Heart Centre, Department of Cardiology, Kuching, Sarawak, Malaysia.

A 59-year-old Iban man with an underlying hypertension and a history of pulmonary tuberculosis experienced an on-off chest pain and reduced effort tolerance for 1 month. He presented to the hospital due to a worsening chest pain.

**FIGURE 1:**
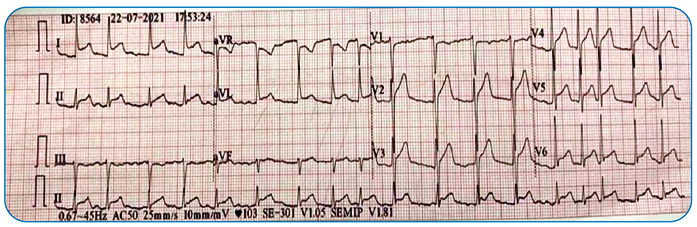
Electrocardiogram showing diffuse ST elevation.

On physical examination, the patient was afebrile. Result of the cardiovascular examination was unremarkable. Consolidation or effusion was not observed on chest radiography. Electrocardiography showed diffuse ST elevation; hence, fibrinolytic therapy was administered (Figure 1). Electrocardiography showed persistent ST elevation without dynamic ischemic changes. The patient’s C-reactive peptide level was high (408.8 mg/L). 

**FIGURE 2:**
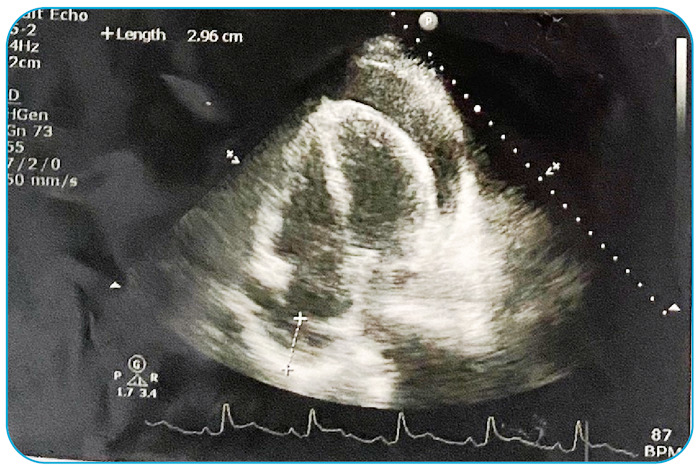
Echocardiography showing pericardial effusion and tamponade signs.

The ST elevation myocardial infarction had reverted to pericarditis. Nonsteroidal anti-inflammatory drug therapy was started but was later switched to high-dose aspirin due to acute kidney injury. Echocardiography revealed pericardial effusion with signs of tamponade (Figure 2). Pericardiocentesis was performed, and the pericardial fluid culture showed *Salmonella* enteritidis. Intravenous ceftriaxone was administered. 

The patient’s condition deteriorated. 

**FIGURE 3:**
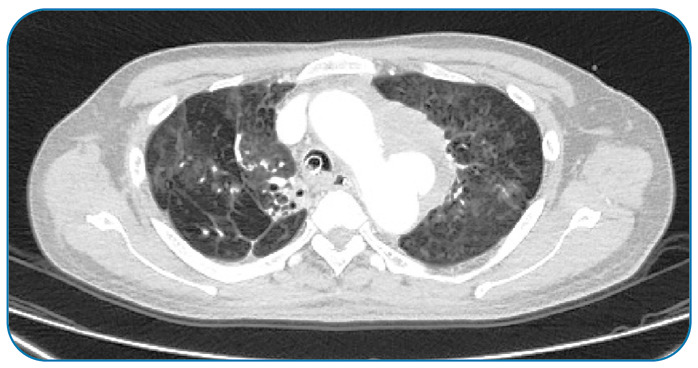
Computed tomography angiogram showing a ruptured aortic arch saccular aneurysm (arrow).

Patient’s computed tomography angiogram showed a ruptured aortic arch saccular aneurysm measuring 1.7×3×4.1 cm, surrounded by a rim of mediastinal hematoma 5.7×6.6×5.3 cm that extended into the left main bronchus (Figure 3). 

The patient was frail to undergo surgical intervention and eventually died. 

Mycotic pseudoaneurysms and massive pericardial effusion due to *Salmonella* infection rarely occur^1^. However, complications are often fatal. Hence, early diagnosis and intervention are crucial to reduce mortality^1^.
